# Expression of basic fibroblast growth factor and its receptor in human pancreatic carcinomas.

**DOI:** 10.1038/bjc.1995.420

**Published:** 1995-10

**Authors:** T. Ohta, M. Yamamoto, M. Numata, S. Iseki, Y. Tsukioka, T. Miyashita, M. Kayahara, T. Nagakawa, I. Miyazaki, K. Nishikawa, Y. Yoshitake

**Affiliations:** Department of Surgery (II), School of Medicine, Kanazawa University, Japan.

## Abstract

**Images:**


British Journal of Cancer (1995) 72, 824-831
© 1995 Stockton Press All rights reserved 0007-0920/95 $12.00

### Expression of basic fibroblast growth factor and its receptor in human pancreatic carcinomas

T Ohta[1], M Yamamoto[2], M Numata[2], S Iseki[2], Y Tsukioka[1], T Miyashita[1], M Kayahara[1], T Nagakawa[1], I Miyazaki[1], K Nishikawa[3] and Y Yoshitake[3]

*Departments of [1]Surgery (II) and [2]Anatomy (I), School of Medicine, Kanazawa University, Takara-machi 13-1, Kanazawa 920, Japan; [3]Department of Biochemistry, Kanazawa Medical University, Uchinada 920-02, Japan.*

**Summary** We examined the expression of basic fibroblast growth factor (FGF) and FGF receptor by immunohistochemistry in 32 human pancreatic ductal adenocarcinomas. Mild to marked basic FGF immunoreactivity was noted in 19 (59.4%) of the 32 tumours examined, and 30 (93.3%) of the tumours exhibited a cytoplasmic staining pattern against FGF receptor. The tumours were divided into two groups according to the proportion of positively stained tumour cells: a low expression group (positive cells $<25\%$) and a high expression group (positive cells $\geqslant 25\%$). No statistically significant difference in tumour size, differentiation, metastases or stage was found between the low and high basic FGF expression groups. However, a significant correlation was found between FGF receptor expression level and the presence of retroperitoneal invasion, lymph node metastasis, and tumour stage. In addition, low FGF receptor expression was significantly associated with a longer post-operative survival as compared with high FGF receptor expression, whereas there was no significant difference in post-operative survival between the low and high basic FGF expression groups. Increased expression of FGF receptor is correlated with the extent of malignancy and post-operative survival in human pancreatic ductal adenocarcinomas. Thus, overexpression of FGF receptor may prove to be a more useful prognostic marker than basic FGF expression level in pancreatic cancer patients.

**Keywords:** basic fibroblast growth factor; fibroblast growth factor receptor; human pancreatic cancer

Members of the fibroblast growth factor (FGF, or heparin-binding growth factor) family are potent mitogens for a wide variety of mesodermal and neuroectodermal cells and have been isolated from a variety of tissue and cell sources including tumour cells (Thomas and Gimenetz-Gallego, 1986; Gospodarowicz *et al.*, 1987). To date, at least nine members have been identified from both normal and tumour tissues, including basic FGF, acidic FGF, the *int*-2 gene product (FGF-3), Kaposi FGF (FGF-4), FGF-5, FGF-6, keratinocyte growth factor (FGF-7), androgen-induced growth factor, and FGF-9 (Klagsburn, 1989; Tanaka *et al.*, 1992; Miyamoto *et al.*, 1993).

Basic FGF is thought to induce fibrosis, angiogenesis, and tumour progression in human gastric carcinomas, renal cell carcinomas, brain tumours, and malignant melanoma through an autocrine mechanism (Becker *et al.*, 1989; Takahashi *et al.*, 1990; Zagzag *et al.*, 1990; Tanimoto *et al.*, 1991; Eguchi *et al.*, 1992). Pancreatic carcinomas exhibit strong stromal reactions, or desmoplasia, and have an aggressive behaviour and poor prognosis (Ohta *et al.*, 1993). Therefore, it is feasible that basic FGF is the factor responsible for desmoplasia and cancer cell proliferation in pancreatic carcinomas. This hypothesis is supported by a study in which basic FGF expression was detected in two human pancreatic carcinoma cell lines (Beauchamp *et al.*, 1990). A recent study (Yamanaka *et al.*, 1993; Leung *et al.*, 1994) has also demonstrated the overexpression of basic FGF in human pancreatic carcinoma tissues. In addition, pancreatic carcinoma cells overexpress the FGF receptor which possesses intrinsic tyrosine kinase activity, raising the possibility that the abundance of basic FGF and its receptor may provide human pancreatic carcinoma cells with a considerable growth advantage (Kobrin *et al.*, 1993; Leung *et al.*, 1994). However, the tissue localisation of basic FGF and its receptor proteins have not been fully elucidated in human pancreatic carcinomas.

We examined the immunohistochemical localisation of basic FGF and its receptor in human pancreatic carcinomas and normal pancreatic tissues at the light and electron microscopic level, and determined the relevance of this growth factor system to malignant transformation and clinical parameters including prognosis.

Correspondence: T Ohta
Received 3 June 1994; revised 2 March 1995; accepted 29 May 1995

### Materials and methods

### *Patients and tissue specimens*

The present study included 32 pancreatic ductal adenocarcinomas surgically resected between 1987 and 1993. All tumours were histologically proven to be pancreatic invasive tubular and/or papillary adenocarcinoma. There were no periampullary tumours or distal bile duct tumours not originating from the pancreatic duct. The patients were 22 men and ten women, ranging from 32 to 77 years of age, with a mean age of 63 years. Normal pancreatic tissues were obtained from two male and three female patients undergoing surgery for gastric cancer with combined resection of the distal pancreas and spleen. The resected specimens with attached peripancreatic lymph nodes and neural plexuses were routinely fixed in 10% neutral-buffered formalin and embedded in paraffin, and cut into 5 mm stepwise tissue sections. Histological findings were evaluated according to the General Rules for Cancer of the Pancreas proposed by the Japanese Pancreatic Society (1986). All of the patients on the study were followed until December 1993. Four patients died within 60 days after surgery because of sepsis and hepato-renal failure, and 22 patients relapsed with carcinoma of the pancreas and died from progressive disease in the liver and/or peritoneum. Two patients died of other or unknown causes and four patients survived.

Three or more representative sections, including areas of associated chronic pancreatitis adjacent to the carcinoma, were used for immunohistochemical staining as described below. In addition, in two selected cases, parallel samples were fixed immediately with 4% paraformaldehyde in 0.1 M phosphate buffer, pH 7.2, for 4 h. The tissue blocks were further rinsed overnight in a phosphate buffer containing 20% sucrose, then cut into 15–20 μm sections on a cryostat and mounted on poly-L-lysine-coated glass slides for immunoelectron microscopy of basic FGF.

*Antibodies*

Monoclonal antibody against human basic FGF was obtained and purified as described previously (Matsuzaki *et al.*, 1989; Yoshitake *et al.*, 1991). This antibody is highly specific for basic FGF from human, bovine and rodent sources, and does not cross-react with acidic FGF. The anti-FGF receptor antibody was a polyclonal antibody raised in rabbits against purified human recombinant FGF receptor (Flg-5) extracellular domain (Austral Biologicals, CA, USA). This polyclonal antibody recognises recombinant human FGF receptor as evidenced by Western analysis (Figure 1).

*Light microscopic immunohistochemistry*

Immunohistochemistry was performed using a three-step indirect immunoperoxidase method (streptavidin–biotin–peroxidase complex) as previously reported (Hughes and Hall, 1993) with a slight modification. Briefly, 4 μm sections were mounted on poly-L-lysine-coated glass slides, air-dried, and deparaffinised with graded xylene and alcohol. For basic FGF staining, protease digestion was carried out using protease K (Boehringer Mannheim Biochemica, Germany) at a concentration of 40 μm $ml^{-1}$ for 5 min at 37°C to facilitate penetration of the primary antibody. Following a phosphate-buffered saline (PBS) rinse, the sections were immersed in absolute methanol containing 0.3% hydrogen peroxide to block endogenous peroxidase activity, and incubated with normal goat serum at a 1:30 dilution for 30 min at room temperature to block non-specific binding. Each primary antibody was diluted in PBS/0.3% bovine serum albumin and used at the predetermined optimal dilution (10 μg $ml^{-1}$). After overnight incubation at 4°C, the sections were rinsed in PBS and incubated for 1 h at room temperature with a biotinylated goat anti-mouse or goat anti-rabbit IgG (Dakopatts, Copenhagen, Denmark). The pcroxidase labelled streptavidin (Dakopatts, Copenhagen, Denmark) was then added for 30 min at room temperature. Reaction products were developed by immersing the sections in a 3.3′-diaminobenzidine tetrahydrochloride solution containing 0.1% hydrogen peroxide. Slides were counterstained lightly with methyl green. In each immunostaining run, the primary antibody was replaced by non-immune normal mouse serum (Dako, Santa Barbara, CA, USA) or PBS as negative controls, which resulted in no detectable staining. Sections from normal skin tissue specimens were used as positive controls which showed positive staining of sweat and sebaceous glands (Hughes and Hall, 1993).

*Immunohistochemical quantification of staining with basic FGF or FGF receptor*

The degree of primary antibody reactivity on individual tissue sections was scored semi-quantitatively (percentage of stained carcinoma cells in the section) by two authors (TO and YT) without knowledge of the patients' outcome or clinicopathological features. Tumours with more than 5% stained cells were defined as positive and all others as negative. The proportion of positively stained tumour cells was subdivided as follows: minimal (+) denotes 5–25% of cells positive, moderate (+ +) denotes 25–50% of cells positive, and marked (+ + +) denotes more than 50% of cells positive. In addition, staining intensity was evaluated visually and each specimen was assigned to one of the follow-

**Figure 1** Western blot analysis of the specificity of anti-FGF receptor polyclonal antibody. The recombinant human FGF receptor (Austral Biologicals, CA, USA) conjugated with BSA (0.1 μg per lane) was electrophoresed, blotted onto a nitrocellulose membrane, and immunoreacted with anti-FGF receptor antibody (**a**) and non-immune normal rabbit serum (**b**) at 1:200 dilution in PBS. As a result, the anti-FGF receptor antibody immunoreacted with recombinant human FGF receptor conjugated with BSA, forming a single major band of approximately 68 kDa. In contrast, non-immune normal rabbit serum showed no reaction with this antigen.

**Figure 2** Light microscopic immunostaining for basic FGF and FGF receptor in normal human pancreas. (**a**) Basic FGF immunoreactivity is present in a heterogenous pattern in acinar cells, and is rarely present in ductal cells (× 140); (**b**) FGF receptor is present in ductal cells and centroacinar cells, however, there is no staining in the acinar cells. Endothelial cells in the stroma (arrows) occasionally show FGF receptor immunoreactivity (× 140).

ing categories: no staining (−), weak staining (W), and strong staining (S).

To determine the relationship between the overexpression of basic FGF or the FGF receptor and the biological behaviour of invasive ductal adenocarcinoma of the pancreas, the 32 patients were classified into two groups according to the proportion of positively stained tumour cells: group 1, patients with no staining or with less than 25% positive tumour cells (low-expression group); group 2, patients with more than 25% positive tumour cells (high-expression group).

*Electron microscopic immunocytochemistry*

Sections immunostained using the three-step indirect immunoperoxidase method described above were post-fixed with 0.5% osmium tetroxide for 20 min at room temperature. After block-staining with uranyl acetate, the sections were dehydrated in graded ethanol, embedded in Epon 812, and cut into ultrathin sections.

*Statistical analysis*

Statistical comparisons on baseline data between the two groups were performed by the chi-square test. The cumulative survival rate was calculated by the Kaplan–Meier method. This was done under the consideration that the number of cases in each group was not large. Statistical analysis of differences between the two groups was made by the log-rank test. The difference was considered to be significant when $P<0.05$.

### Results

*Light microscopic immunohistochemistry for basic FGF*

In most sections of normal pancreas, moderate basic FGF immunoreactivity was present in a heterogeneous pattern in acinar cells. It was most important at the basal aspect of the acinar cells (Figure 2a). Relatively weak cytoplasmic staining of some intralobular and interlobular duct cells was also seen. However, immunostaining was rarely present in islet cells or stromal cells.

Nineteen of the 32 pancreatic ductal adenocarcinomas (59.4%) showed minimal to marked immunoreactivity for basic FGF (Table I). Eleven of the 19 positively stained tumours exhibited cytoplasmic immunoreactivity (Figure 3a,b), while the other eight showed predominantly nuclear immunoreactivity, a phenomenon which was not observed in the normal pancreas (Figure 3c). Twelve of the adenocarcinomas (40.6%) showed little or no immunostaining in the carcinoma cells. However, intense basic FGF immunoreactivity was seen in many surrounding stromal cells including fibroblasts and macrophages (Figure 3d). In areas of associated chronic pancreatitis, there was a considerable increase in basic FGF immunoreactivity in the atrophied acinar and ductal cells in comparison with normal pancreas.

*Light microscopic immunohistochemistry for FGF receptor*

Most sections of normal pancreas showed intense cytoplasmic staining for FGF receptor in intralobular, interlobular

**Figure 3** Light microscopic immunostaining for basic FGF in human pancreatic ductal adenocarcinoma. (**a**) and (**b**) Intense cytoplasmic immunoreactivity for basic FGF is present not only in carcinoma cells but also in the surrounding fibroblasts (× 70 and × 210 respectively). Endothelial cells in the stroma (arrow) also react with basic FGF; (**c**) Some tumours exhibit a predominant nuclear immunoreactivity (× 140); (**d**) There is no staining in carcinoma cells. However, the surrounding stromal cells, including fibroblasts and macrophages, show intense basic FGF immunoreactivity (× 112).

**Table I** Immunostaining of human pancreatic cancer specimens with anti-basic FGF and anti-FGF receptor antibodies

| | *Basic FGF* | | | *FGF receptor* | |
|---|---|---|---|---|---|
| *Case Number* | *Stained proportion* | *Staining intensity* | *Staining pattern* | *Stained proportion* | *Staining intensity* |
| 1 | +++ | W | N | +++ | S |
| 2 | ++ | W | C | ++ | S |
| 3 | ++ | S | C | ++ | S |
| 4 | +++ | S | N | +++ | S |
| 5 | ++ | W | C | +++ | S |
| 6 | ++ | W | C | +++ | S |
| 7 | +++ | S | N | ++ | S |
| 8 | +++ | S | N | ++ | S |
| 9 | +++ | S | N | +++ | S |
| 10 | ++ | W | C | +++ | S |
| 11 | ++ | W | C | +++ | S |
| 12 | +++ | W | C | +++ | S |
| 13 | ++ | W | C | +++ | S |
| 14 | ++ | S | N | + | S |
| 15 | ++ | W | C | + | S |
| 16 | ++ | S | N | + | S |
| 17 | ++ | S | N | + | S |
| 18 | + | W | C | +++ | S |
| 19 | + | W | C | ++ | S |
| 20 | – | – | | ++ | S |
| 21 | – | – | | +++ | S |
| 22 | – | – | | +++ | S |
| 23 | – | – | | ++ | S |
| 24 | – | – | | ++ | S |
| 25 | – | – | | ++ | S |
| 26 | – | – | | ++ | S |
| 27 | – | – | | ++ | S |
| 28 | – | – | | +++ | S |
| 29 | – | – | | ++ | S |
| 30 | – | – | | + | S |
| 31 | – | – | | – | – |
| 32 | – | – | | – | – |

Stained proportion: –, all cells negative or $<5\%$ of cells positive; +, 5–20% of cells positive; ++, 25–50% of cells positive; +++, 50–100% of cells positive. Staining intensity: –, no staining; W, weak intensity; S, strong intensity. Staining pattern: N, nuclear staining type; C, cytoplasmic staining type.

**Figure 4** Light microscopic immunostaining for FGF receptor in human pancreatic ductal adenocarcinomas. FGF receptor is found on both the cell surface and in the cytoplasm, and is especially prominent at the apical surfaces of carcinoma cells (**a**, ×70; **b**, ×182).

and main pancreatic duct cells and weak cytoplasmic staining of centroacinar cells and intercalated ducts (Figure 2b). However, there was no staining in the acinar cells, islet cells or surrounding stromal cells.

Thirty of the 32 pancreatic ductal adenocarcinomas (93.8%) showed minimal to marked immunoreactivity for FGF receptor (Table I). The staining intensity in the tumours varied from specimen to specimen, as well as from area to area within the same specimen. In these positive cells, FGF receptor was found on both the cell surface and in the cytoplasm, and was especially prominent at the apical surfaces (Figure 4). There was no or only weak immunostaining in the stromal cells surrounding the carcinoma cells. However, in some cases, stromal cells in the infiltrative margin of the tumours showed moderate to strong immunoreactivity. In the area of associated chronic pancreatitis, there was a considerable increase in FGF receptor immunoreactivity in the atrophied acinar and ductal cells in comparison with a normal pancreas.

### *Immunoelectron microscopy for basic FGF*

Most spindle-shaped cells positive for basic FGF were identified as fibroblasts (Figure 5a). The immunoreactivity for basic FGF was located in the cytosol (cytoplasmic matrix), and was especially prominent in the cytosol adjacent to the rough endoplasmic reticulum. Carcinoma cells also showed basic FGF immunoreactivity in the cytosol and rarely in the rough endoplasmic reticulum and Golgi apparatus (Figure 5b). No distinct staining was detected in the nucleus in the two specimens examined.

### *Relationship between basic FGF or FGF receptor expression levels and clinicopathological features in pancreatic cancers*

No statistically significant difference in tumour size, tumour location, anterior capsular invasion, retroperitoneal invasion, histological differentiation, presence of lymph node metastases, presence of liver metastases, or tumour stage were found between the low and high basic FGF expression groups (Table II). In contrast, significant difference in retroperitoneal invasion ($P<0.05$), lymph node metastasis ($P<0.05$), and tumour stage ($P<0.01$) was found between the low and high FGF receptor groups (Table II).

### *Survival analysis*

Survival data were available for 28 of the 32 patients. There was no significant difference in post-operative survival between the low and high basic FGF expression groups (Figure 6). In contrast, low FGF receptor expression was associated with longer post-operative survival as compared with high FGF receptor expression and this difference was statistically significant ($P<0.01$), although the low FGF receptor expression group represented only a small subgroup of the total population (Figure 7).

### Discussion

The detection of small pancreatic cancers in Japan has been increasing with improvements in diagnostic methods and the a b

**Figure 5** Immunoelectron micrograph for basic FGF in human pancreatic ductal adenocarcinoma. (**a**) Immunoreactivity in a fibroblast adjacent to carcinoma cells is mainly located in the cytosol adjacent to the rough endoplasmic reticulum (× 8800); (**b**) Carcinoma cell with intense immunoreactivity in the cytosol, and rarely in the rough endoplasmic reticulum and Golgi apparatus (× 16 000).

discovery of tumour markers for pancreatic cancer (Ariyama *et al*., 1990; Satake *et al*., 1991). However, even if pancreatic ductal adenocarcinomas, excluding an intraductal variant of mucin-producing pancreatic tumour (Morohoshi *et al*., 1989), are detected early and completely resected, the incidence of recurrence after pancreatectomy is high and the prognosis is poor (Kayahara *et al*., 1993; Ohta *et al*., 1993). This may be due to the aggressive biological behaviour of this cancer.

Recently, various prognostic factors for pancreatic cancers, including DNA nuclear content analysis, argyrophilic nucleolar organiser region (Ag-NOR) counts, and the presence or absence of overexpression of various proto-oncogenes, growth factors, and their receptors have been investigated. However, there have been only a few reports of reliable prognostic factors for pancreatic cancers (Alanen *et al*., 1990; Motojima *et al*., 1991; Tian *et al*., 1992; Nakamori *et al*., 1993). Therefore, it is essential to examine resected specimens for features that might correlate with survival. These features, if identified, would be a guide to prognosis after operation.

Basic FGF has been implicated in tumour angiogenesis through its ability to stimulate the growth of endothelial cells (Folkman and Klagsburn, 1987). Additionally, this growth factor stimulates fibroblast and epithelial cell growth (Rizzino *et al*., 1986; Ristow and Messmer, 1988). Basic FGF mediates its biological effects by binding to a high-affinity cell surface receptor (FGF receptor) containing an intracellular tyrosine kinase domain (Fresel *et al*., 1986; Olwin and Hauschka, 1989; Klagsburn and Baird, 1991). Schweigerer *et al*. (1987*b*) reported that basic FGF is an autocrine growth factor for human embryonal rhabdomyosarcoma cells. In addition, human gastric cancers, gliomas, meningiomas and renal cell carcinomas have been reported to express basic FGF mRNA (Takahashi *et al*., 1990; Zagzag *et al*., 1990; Tanimoto *et al*., 1991; Eguchi *et al*., 1992), and Kaposi's sarcoma cells have been reported to release basic FGF into their medium (Ensoli *et al*., 1989). However, basic FGF lacks a typical signal peptide region which facilitates secretion (Gospodarowicz *et al*., 1987) and its release mechanism remains unknown. Cell lysis or leakage may be involved in the release of basic FGF as the existence of similar mechanisms has been proposed for interleukin-1, another growth factor that lacks a signal peptide (Auron *et al*., 1984; March *et al*., 1985; Schweigerer *et al*., 1987*a*; Lemoine *et al*., 1993).

Previous studies have demonstrated that human pancreatic carcinoma cell lines overexpress basic FGF and the FGF receptor (Beauchamp *et al*., 1990; Lemoine *et al*., 1993). In addition, a recent study has indicated that there are increased levels of basic FGF and FGF receptor in human pancreatic cancers as compared with normal human pancreatic tissues, using immunohistochemical staining, northern blotting, and *in situ* hybridisation (Kobrin *et al*., 1993; Yamanaka *et al*., 1993; Leung *et al*., 1994). In the present study, we demonstrated the presence of basic FGF and FGF receptor expression in human pancreatic cancers and normal pancreatic tissues by immunocyto- and immunohistochemistry. In the normal pancreas, moderate to marked basic FGF immunoreactivity was present in a heterogeneous pattern at the basal aspect of acinar cells, and intense cytoplasmic FGF receptor immunoreactivity was seen in intralobular, interlobular and main pancreatic duct cells. Additionally, in the human pancreatic cancers minimal to marked basic FGF immunoreactivity was noted in 19 (59.4%) of the 32 tumours and 30 (93.8%) tumours showed minimal to marked cytoplasmic staining for FGF receptor. This suggests that there is concomitant expression of basic FGF and FGF receptor in pancreatic ductal adenocarcinomas, which may allow for excessive autocrine growth stimulation. Furthermore, eight (25%) tumours had nuclear staining for basic FGF, supporting the concept of an intracellular stimulating effect like that of sis protein (Yamamoto *et al*., 1991; Nakanishi *et al*., 1992), i.e. the presence of basic FGF protein in the nucleus has raised the possibility of specific nuclear functions for this molecule in addition to signalling at the cell surface (Mason, 1994). Thus, tumour-derived basic FGF may play a role as a

**Table II** Relationship between basic FGF or FGF receptor expression level and clinicopathological features in human pancreatic cancers

| Variables[a] | Basic FGF Low expression group (%) | Basic FGF High expression group (%) | FGF receptor Low expression group (%) | FGF receptor High expression group (%) |
|---|---|---|---|---|
| No. of patients | 15 | 17 | 7 | 25 |
| Tumour size | | | | |
| ⩽3.0 cm | 3 (20) | 4 (24) | 3 (43) | 4 (16) |
| >3.0 cm | 12 (80) | 13 (76) | 4 (57) | 21 (84) |
| Tumour location | | | | |
| Head | 13 (87) | 11 (65) | 4 (57) | 19 (76) |
| Body and tail | 2 (13) | 6 (35) | 3 (43) | 6 (24) |
| Anterior capsular invasion | | | | |
| Negative | 8 (53) | 7 (41) | 4 (57) | 11 (44) |
| Positive | 7 (47) | 10 (59) | 3 (43) | 14 (56) |
| Retroperitoneal invasion | | | | |
| Negative | 3 (20) | 3 (18) | 4 (57) | 2 (8) |
| Positive | 12 (80) | 14 (82) | 3 (43)[b] | 23 (92)[b] |
| Histological differentiation | | | | |
| Well/moderately | 14 (93) | 15 (88) | 7 (100) | 22 (88) |
| Poorly | 1 (7) | 2 (12) | 0 | 3 (12) |
| Lymph node metastasis | | | | |
| Negative | 3 (20) | 1 (6) | 3 (43) | 1 (4) |
| Positive | 12 (80) | 16 (94) | 4 (57)[b] | 24 (96)[b] |
| Liver metastasis | | | | |
| Negative | 12 (80) | 13 (76) | 6 (86) | 19 (76) |
| Positive | 3 (20) | 4 (24) | 1 (14) | 6 (24) |
| Tumour stage | | | | |
| I/II | 2 (13) | 2 (12) | 4 (57) | 0 |
| III/IV | 13 (87) | 15 (88) | 3 (43)[b] | 25 (100)[b] |

[a]Histological findings are evaluated according to the *General Rules for Cancer of the Pancreas* proposed by the Japanese Pancreatic Society (1986). [b]Analysed by chi-square test. $P<0.05$.

**Figure 6** Cumulative survival curves of patients with resected pancreatic ductal adenocarcinomas, subdivided according to the basic FGF expression level. ○—○, High-expression group (positive cells ⩾25%); ●—●, low-expression group (positive cells <25%). There is no significant difference in post-operative survival between the low and high basic FGF expression groups.

**Figure 7** Cumulative survival curves of patients with resected pancreatic ductal adenocarcinomas, subdivided according to the FGF receptor expression level. ○—○, High-expression group (positive cells ⩾25%); ●—●, low-expression group (positive cells <25%). Low FGF receptor expression is significantly associated with longer post-operative survival ($P<0.01$).

potent mitogen in tumour growth and desmoplastic response; however, the main function of this protein in human pancreatic ductal cancers may not be to promote angiogenesis because pancreatic ductal cancers are almost invariably hypovascular. In contrast, brain tumours are known to have more intense neovascularisation than other tumours and produce basic FGF as a potent angiogenic mediator (Li *et al.*, 1994). Additionally, although 13 tumours (40.6%) showed no basic FGF immunoreactivity, intense basic FGF immunoreactivity was seen in the adjacent fibroblasts in all basic FGF negative tumours, and 11 of 13 basic FGF negative tumours (84.6%) displayed mild to marked immunoreactivity to the FGF receptor. These findings suggest that basic FGF-negative carcinoma cells could be targets for paracrine growth control by basic FGF produced by stromal components. This hypothesis is supported by several experimental studies suggesting the importance of contacts between tumour cells and fibroblasts (Tanaka *et al.*, 1988; Coucke *et al.*, 1992; Gartner *et al.*, 1992).

In the present study, high levels of FGF receptor expression was associated with the presence of retroperitoneal invasion and lymph node metastasis, and with advancing tumour stage, although no statistically significant difference in variable clinicopathological factors was found between the low and high basic FGF expression groups. In addition, low FGF receptor expression was significantly associated with longer post-operative survival, whereas there was no significant difference in post-operative survival between the low and high basic FGF expression groups. Thus, overexpression of FGF receptor may prove to be a more useful prognostic marker than basic FGF expression in pancreatic cancer patients. However, a recent study (Yamanaka *et al.*,

1993) has shown that overexpression of basic FGF is associated with poor prognosis, although almost all the patients had a poor prognosis and died within 3 years of surgery. Further studies with a large number of patients, including a multivariate analysis, are needed to determine whether expression of basic FGF or of the FGF receptor is a better prognostic marker for patients with completely resected adenocarcinoma of the pancreas.

### References

ALANEN KA, JOENSUU H, KLEMI PJ AND NEVALAINEN TJ. (1990). Clinical significance of nuclear DNA content in pancreatic carcinoma. *J. Pathol.*, **160**, 313–320.

ARIYAMA J, SUYAMA M, OGAWA K, IKARI T, NAGAIWA J, FUJII D AND TSUCHIYA A. (1990). The detection and prognosis of small pancreatic carcinoma. *Int. J. Pancreatol.*, **17**, 37–47.

AURON PE, WEBB AC, ROSENWASSER LJ, MUCCI SF, RICH A, WOLFF SM AND DINARELLO CA. (1984). Nucleotide sequence of human monocyte interleukin I precursor cDNA. *Proc. Natl Acad. Sci. USA*, **81**, 7907–7911.

BEAUCHAMP RD, LYONS RM, YANG EY AND MOSES HL. (1990). Expression of and response to growth regulatory peptides by two human pancreatic carcinoma cell lines. *Pancreas*, **5**, 369–380.

BECKER D, MEIER CB AND HERLYN M. (1989). Proliferation of human malignant melanomas is inhibited by anti-sense oligodeoxynucleotides targeted against basic fibroblast growth factor. *EMBO J.*, **8**, 3685–3691.

COUCKE P, DELEVAL L, LEYH P, BONJEAN K, SIWEK B, NOEL A, DEPAUW-GILLET MC, PAULUS JM, BASSLEER R AND FOIDART JM. (1992). Influence of laminine or fibroblasts upon colony formation in the mouse by B-16 melanoma cell spheroids: a morphometric analysis. *In vivo*, **6**, 119–124.

EGUCHI J, NOMATA K, KANDA S, IGAWA T, TAIDE M, KOGA S, MATSUYA F, KANETANI H AND SAITO Y. (1992). Gene expression and immunohistochemical localization of basic fibroblast growth factor in renal cell carcinoma. *Biochem. Biophys. Res. Commun.*, **183**, 937–944.

ENSOLI B, NAKAMURA S, SALAHUDDIN Z, BIBERFELD P, LARSSON L, BEAVER B, WONG-STAAL F AND GALLO RC. (1989). AIDS-Kaposi's sarcoma-derived cells express cytokines with autocrine and paracrine growth effects. *Science*, **243**, 223–226.

FOLKMAN J AND KLAGSBURN M. (1987). Angiogenic factors. *Science*, **235**, 442–447.

FRESEL R, BURGESS WH, MEHLMAN T AND MACIAG T. (1986). The characterization of the receptor for endothelial cell growth factor by covalent ligand attachment. *J. Biol. Chem.*, **261**, 7581–7584.

GARTNER M, WILSON L AND DOWDLE EB. (1992). Fibroblast-dependent tumorigenicity of melanoma xenograft in athymic mice. *Int. J. Cancer*, **51**, 788–791.

GOSPODAROWICZ D, NEUFELD G AND SCHWEIGERER L. (1987). Fibroblast growth factor: structural and biological properties. *J. Cell. Physiol.*, **5** (suppl.), 15–26.

HUGHES SE AND HALL PA. (1993). Immunolocalization of fibroblast growth factor receptor I and its ligands in human tissues. *Lab. Invest.*, **69**, 173–182.

JAPANESE PANCREATIC SOCIETY. (1986). *General Rules for Surgery and Pathological Studies on Cancer of the Pancreas*, 3rd edn. Kanehara: Tokyo.

KAYAHARA M, NAGAKAWA T, UENO K, OHTA T, TAKEDA T AND MIYAZAKI I. (1993). An evaluation of radical resection for pancreatic cancer based on the mode of recurrence as determined by autopsy and diagnostic imaging. *Cancer*, **72**, 2118–2123.

KLAGSBURN M. (1989). The fibroblast growth factor family: structural and biological properties. *Prog. Growth Factor Res.*, **1**, 207–235.

KLAGSBURN M AND BAIRD A. (1991). A dual receptor system is required for basic fibroblast growth factor activity. *Cell*, **67**, 229–231.

KOBRIN MS, YAMANAKA Y, FRIESS H, LOPEZ ME AND KORC M. (1993). Aberrant expression of type I fibroblast growth factor receptor in human pancreatic adenocarcinomas. *Cancer Res.*, **53**, 4741–4744.

LEMOINE NR, LEUNG HY, BARTON CM, HUGHES CM, KLOPPEL G AND GULLICK WJ. (1993). Autocrine growth control of pancreatic cancer. *Int. J. Pancreatol.*, **14**, 69–70.

LEUNG HY, HUGHES CM, KLOPPEL G, WILLIAMSON RCN AND LEMOINE NR. (1994). Localisation of expression of fibroblast growth factors and their receptors in pancreatic adenocarcinoma by in situ hybridisation. *Int. J. Oncol.*, **4**, 1219–1223.

LI VW, FOLKERTH RD, WATANABE H, YU C, RUPNICK M, BARNES P, SCOTT RM, BLACK PM, SALLAN SE AND FOLKMAN J. (1994). Microvessel count and cerebrospinal fluid basic fibroblast growth factor in children with brain tumors. *Lancet*, **344**, 82–86.

MARCH CJ, MOSLEY B, LARSEN A, CERRETTI DP, BRAEDT G, PRICE V, GILLIS S, HENNEY CS, KRONHEIM SR, GRABSTEIN K, CONLON PJ, HOPP TP AND COSMAN D. (1985). Cloning, sequence and expression of two distinct human interleukin-I complementary DNAs. *Nature*, **315**, 641–647.

MASON IJ. (1994). The ins and outs of fibroblast growth factors. *Cell*, **78**, 547–552.

MATSUZAKI K, YOSHITAKE Y, MATUSO Y, SASAKI H AND NISHIKAWA K. (1989). Monoclonal antibodies against heparin-binding growth factor II/basic growth factor that block its biological activity: invalidity of the antibodies for tumor angiogenesis. *Proc. Natl Acad. Sci. USA*, **86**, 9911–9915.

MIYAMOTO ML, NARUO K, SEKO C, MATSUMOTO S, KONDO T AND KUROKAWA T. (1993). Molecular cloning of a novel cytokine cDNA encoding the ninth member of the fibroblast growth factor family, which has a unique secretion property. *Mol. Cell. Biol.*, **13**, 4251–4259.

MOROHOSHI T, KANDA M, ASANUMA K AND KLOPPEL G. (1989). Intraductal papillary neoplasms of the pancreas: a clinicopathologic study of six patients. *Cancer*, **64**, 1329–1335.

MOTOJIMA K, TSUNODA T, KANEMATSU T, NAGATA Y, URANO T AND SHIKU H. (1991). Distinguishing pancreatic carcinoma from other periampullary carcinomas by analysis of mutations in the Kirsten-ras oncogene. *Ann. Surg.*, **214**, 657–662.

NAKAMORI S, ISHIKAWA O, OHIGASHI H, IMAOKA S, SASAKI Y, KAMEYAMA M, KABUTO T, FURUKAWA H, IWANAGA T AND KIMURA N. (1993). Clinicopathological features and prognostic significance of nucleoside diphosphate kinase/nm23 gene product in human pancreatic exocrine neoplasms. *Int. J. Pancreatol.*, **14**, 125–134.

NAKANISHI Y, KIHARA K, MIZUNO K, MASAMUNE U, YOSHITAKE Y AND NISHIKAWA K. (1992). Direct effect of basic fibroblast growth factor on gene transcription in a cell-free system. *Proc. Natl Acad. Sci. USA*, **89**, 5216–5220.

OHTA T, NAGAKAWA T, UENO K, KAYAHARA M, MORI K, KOBAYASHI H, TAKEDA T AND MIYAZAKI I. (1993). The mode of lymphatic and local spread of pancreatic carcinomas less than 4.0 cm in size. *Int. Surg.*, **78**, 208–212.

OLWIN BB AND HAUSCHKA SD. (1989). Cell type and tissue distribution of the fibroblast growth factor receptor. *J. Cell. Biochem.*, **39**, 443–454.

RISTOW HJ AND MESSMER TO. (1988). Basic fibroblast growth factor and insulin-like growth factor I are strong mitogens for cultured mouse keratinocytes. *J. Cell. Physiol.*, **137**, 277–284.

RIZZINO A, RUFF E AND RIZZINO H. (1986). Induction and modulation of anchorage-independent growth by platelet-derived growth factor, fibroblast growth factor, and transforming growth factor-$\beta$. *Cancer Res.*, **46**, 2816–2820.

SATAKE K, CHUNG YS, UMEYAMA K, TAKEUCHI T AND KIM YS. (1991). The possibility of diagnosing small pancreatic cancer (less than 4.0 cm) by measuring various serum tumor markers. *Cancer*, **68**, 149–152.

SCHWEIGERER L, NEUFELD G, FRIEDMAN J, ABRAHAM JA, FIDDES JC AND GOSPODAROWICZ D. (1987*a*). Capillary endothelial cells express basic fibroblast growth factor, a mitogen that promotes their own growth. *Nature*, **325**, 257–259.

SCHWEIGERER L, NEUFELD G, MERGIA A, ABRAHAM JA, FIDDES JC AND GOSPODAROWICZ D. (1987*b*). Basic fibroblast growth factor in human rhabdomyosarcoma cells: implications for the proliferation and neovascularization of myoblast-derived tumors. *Proc. Natl Acad. Sci. USA*, **84**, 842–846.

TAKAHASHI JA, MORI H, FUKUMOTO M, IGARASHI K, JAYE M, ODA Y, KIKUCHI H AND HATANAKA M. (1990). Gene expression of fibroblast growth factors in human gliomas and meningiomas: demonstration of cellular sources of basic fibroblast growth factor mRNA and peptide in tumor tissues. *Proc. Natl Acad. Sci. USA*, **87**, 5710–5715.

TANAKA A, MIYAMOTO K, TAKEDA N, SATO M, MATSUO H AND MATSUMOTO K. (1992). Cloning and characterization of an androgen-induced growth factor essential for the androgen-dependent growth of mouse mammary carcinoma cells. *Proc. Natl Acad. Sci. USA*, **89**, 8928–8932.

TANAKA H, MORI Y, ISHII H AND AKEDO H. (1988). Enhancement of metastatic capacity of fibroblast-tumor cell interaction in mice. *Cancer Res.*, **48**, 1456–1459.

TANIMOTO H, YOSHIDA K, YOKOZAKI H, YASUI W, NAKAYAMA H, ITO H, OHMA K AND TAHARA E. (1991). Expression of basic fibroblast growth factor in human gastric carcinomas. *Virchows Arch. B Cell Pathol.*, **61**, 263–267.

THOMAS KA AND GIMENETZ-GALLEGO G. (1986). Fibroblast growth factors: broad spectrum mitogens with potent angiogenic activity. *Trends Biochem. Sci.*, **11**, 81–84.

TIAN FG, APPERT HE, UYLERS J AND HOWARD JM. (1992). Prognostic value of serum CA 19-9 levels in pancreatic adenocarcinoma. *Ann. Surg.*, **215**, 350–355.

YAMAMOTO N, MATSUTANI S, YOSHITAKE Y, NISHIKAWA K AND NISHIKAWA K. (1991). Immunohistochemical localization of basic fibroblast growth factor in A431 human epidermoid carcinoma cells. *Histochemistry*, **96**, 479–485.

YAMANAKA Y, FRIESS H, BUCHLER M, BEGER HG, UCHIDA E, ONDA M, KOBRIN MS AND KORC M. (1993). Overexpression of acidic and basic fibroblast growth factors in human pancreatic cancer correlates with advanced tumor stage. *Cancer Res.*, **53**, 5289–5296.

YOSHITAKE Y, MATSUZAKI K AND NISHIKAWA K. (1991). Derivation of monoclonal antibody to basic fibroblast growth factor and its application. *Methods Enzymol.*, **198**, 148–157.

ZAGZAG D, MILLER DC, SATO Y, RIFKIN DB AND BURSTEIN DE. (1990. Immunohistochemical localization of basic fibroblast growth factor in astrocytomas. *Cancer Res.*, **50**, 7393–7398.


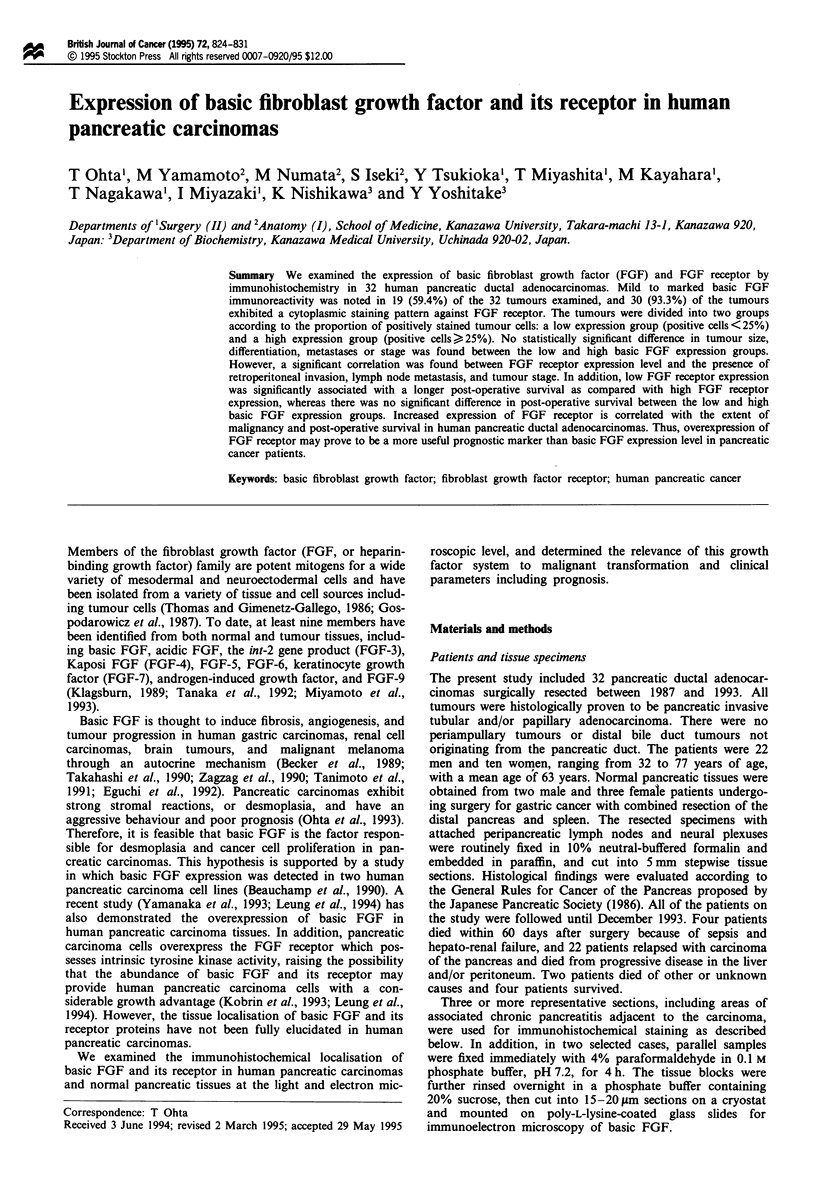

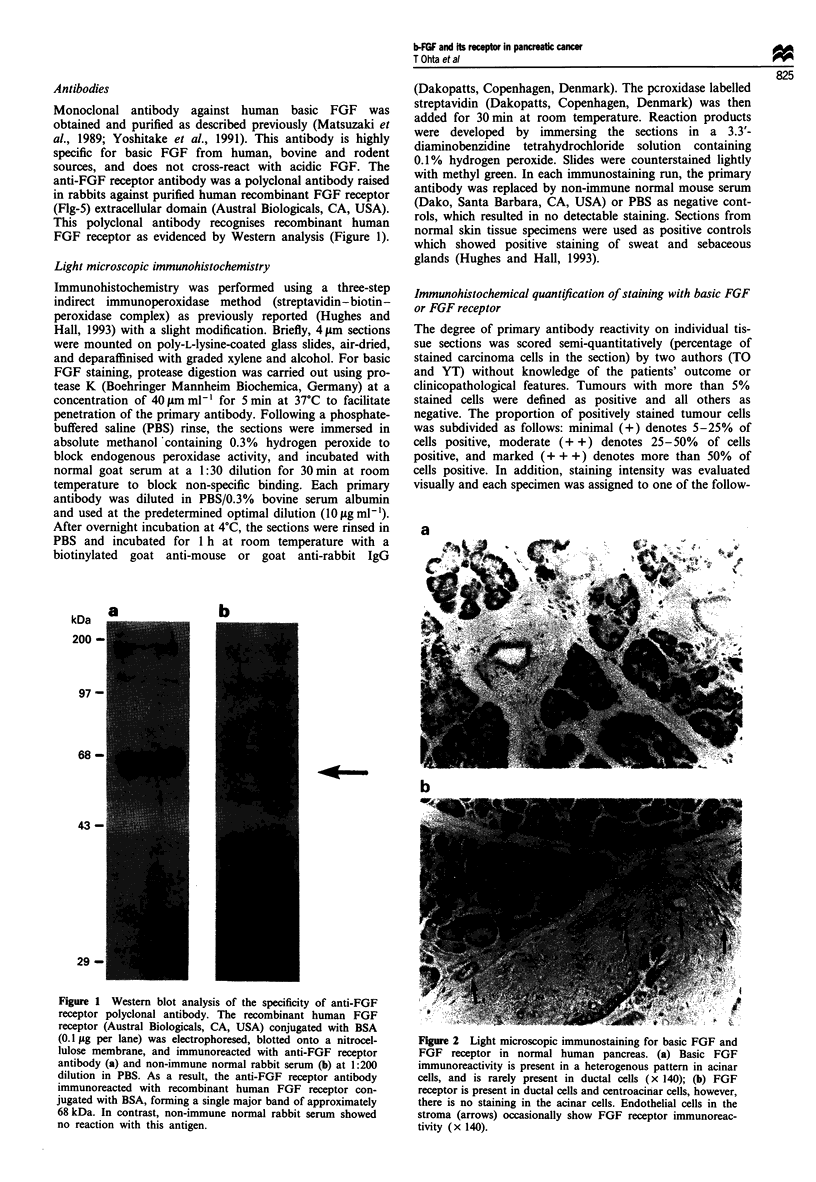

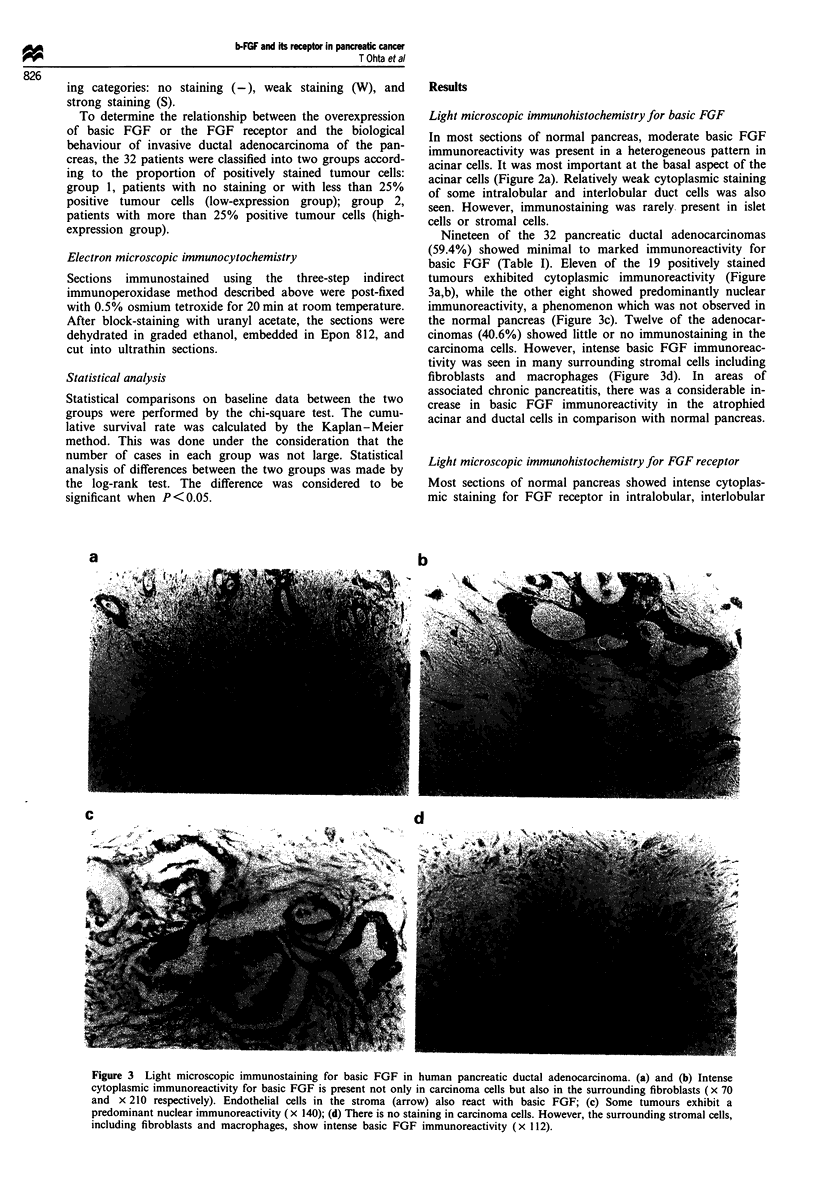

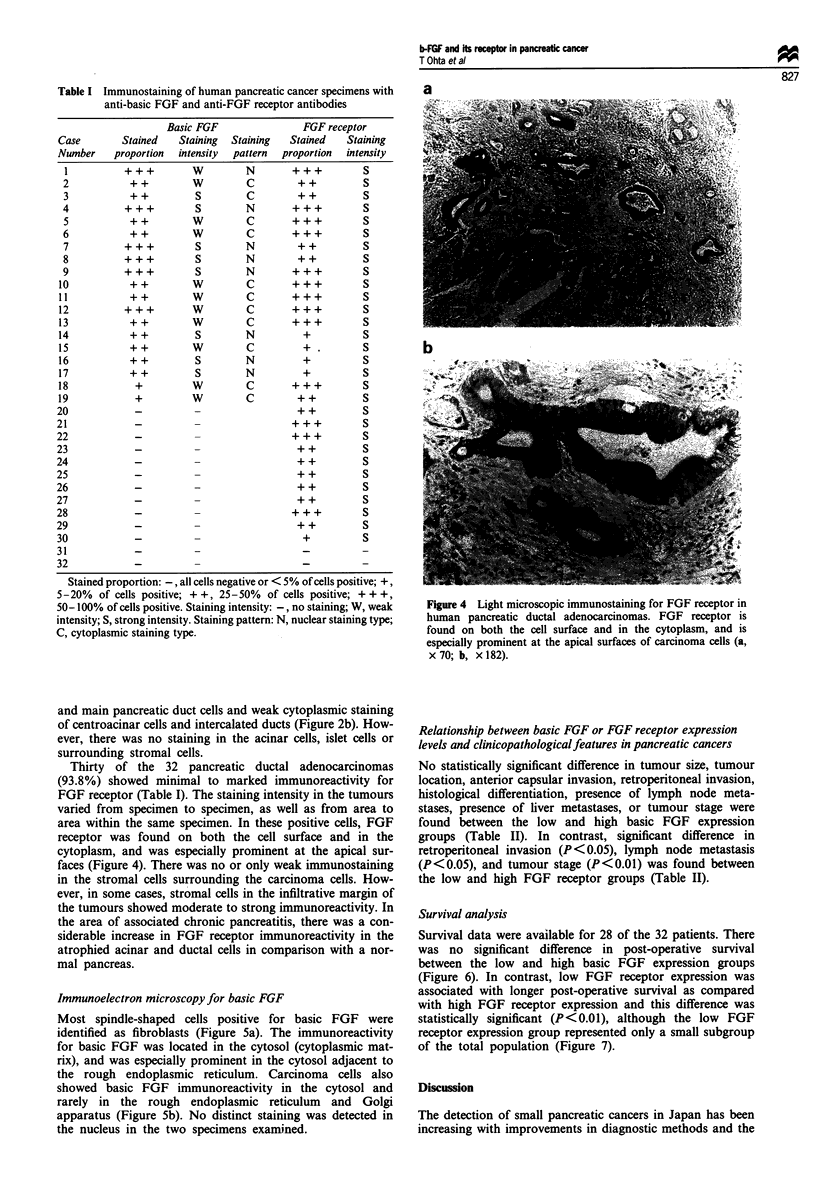

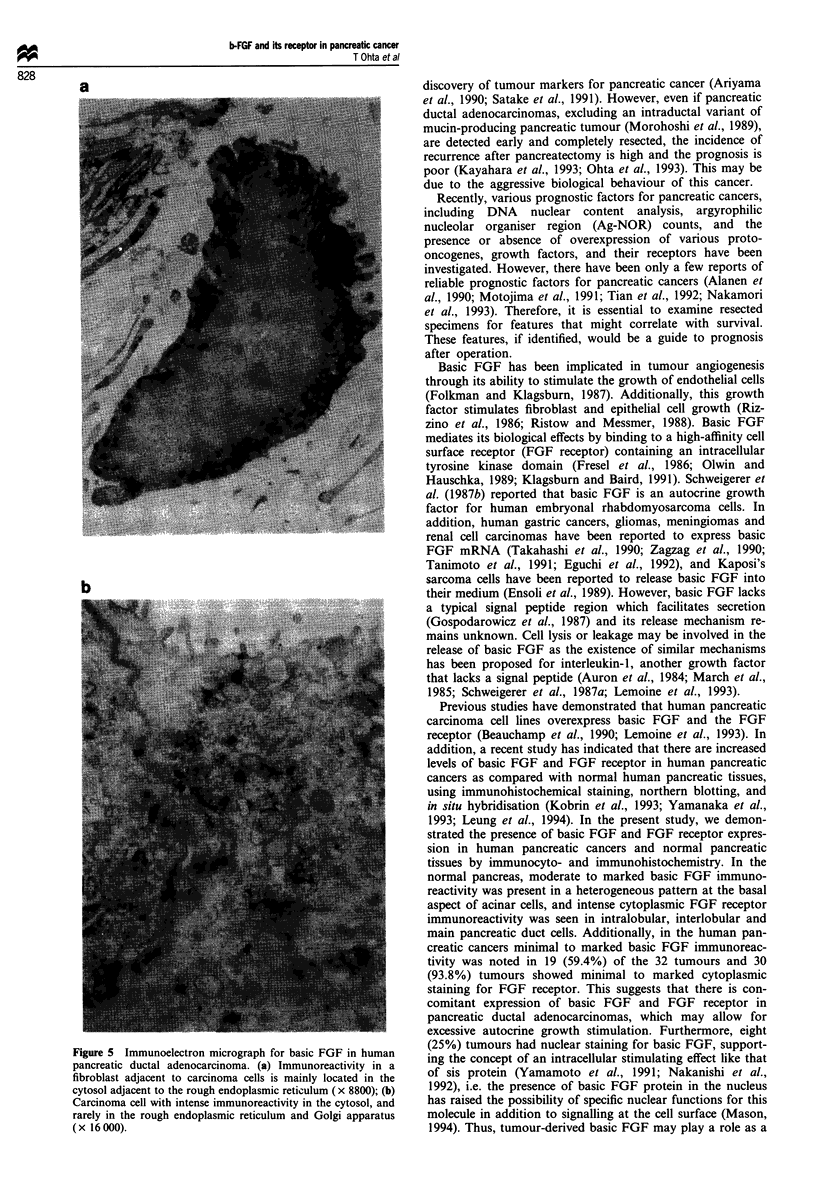

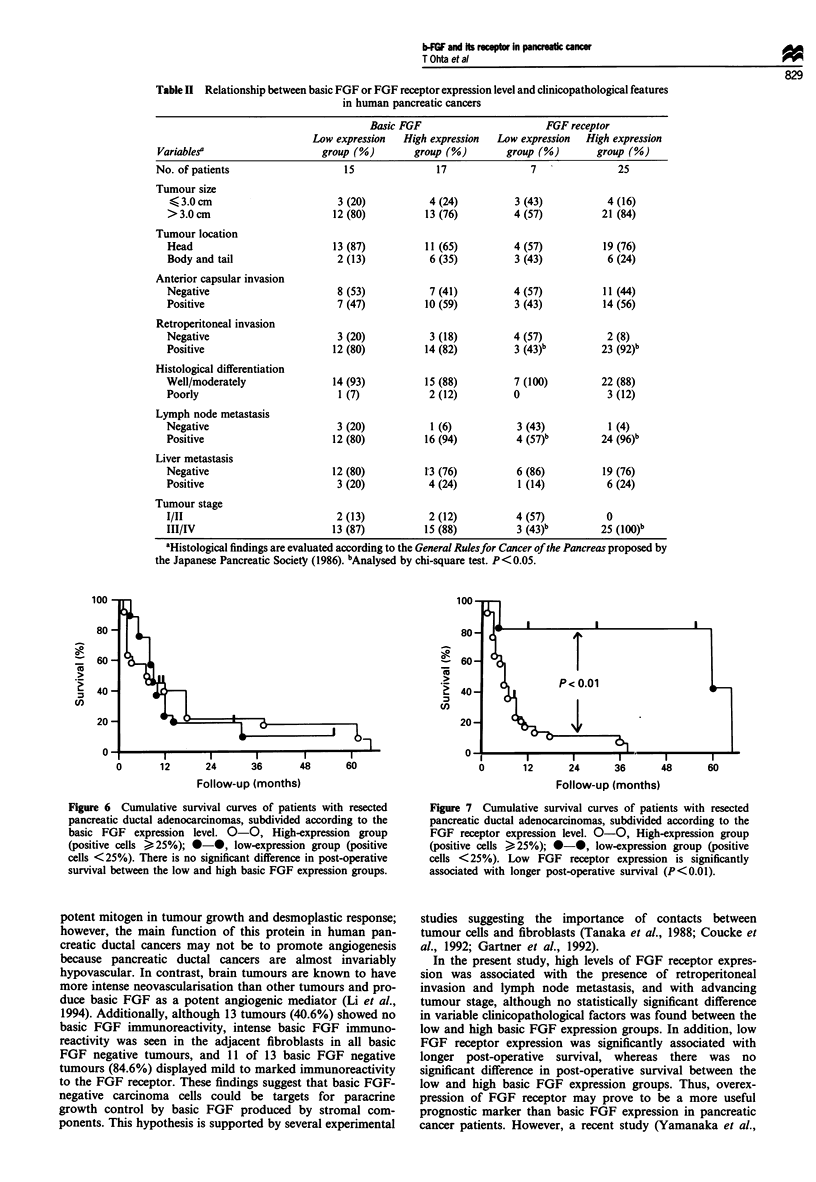

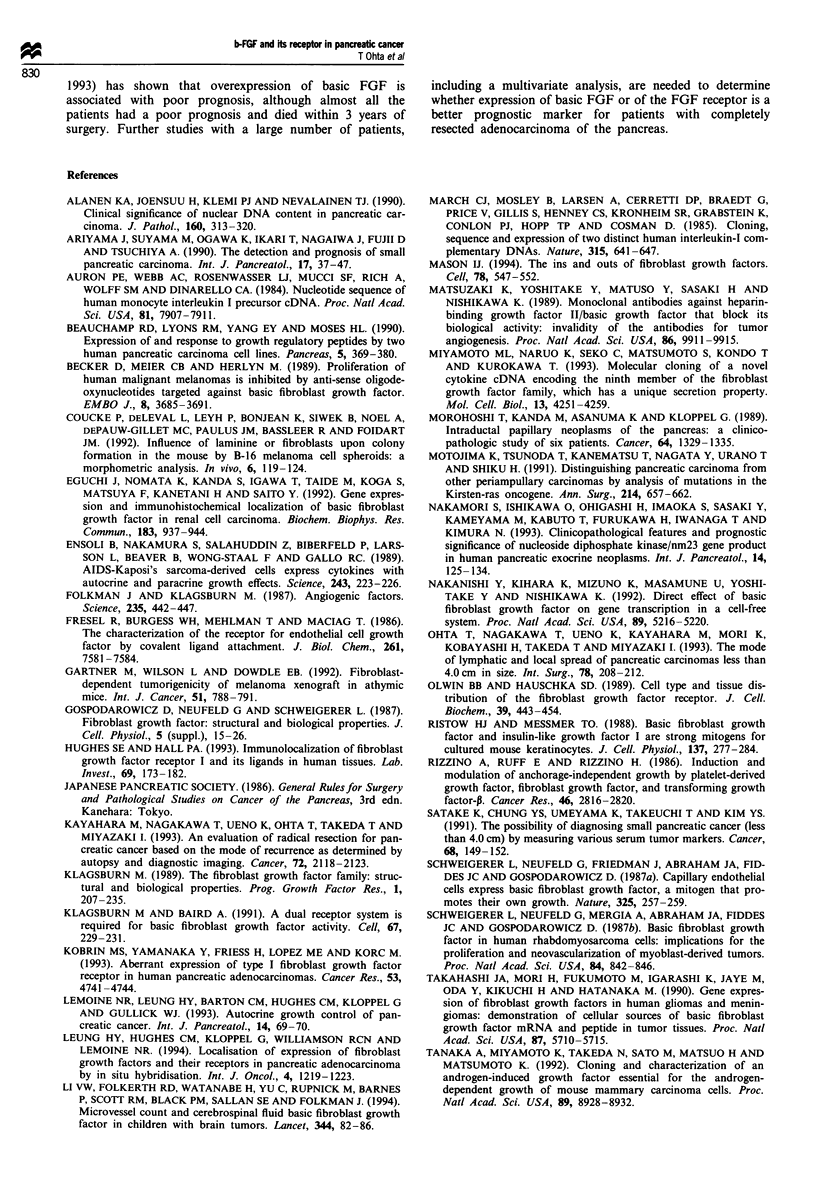

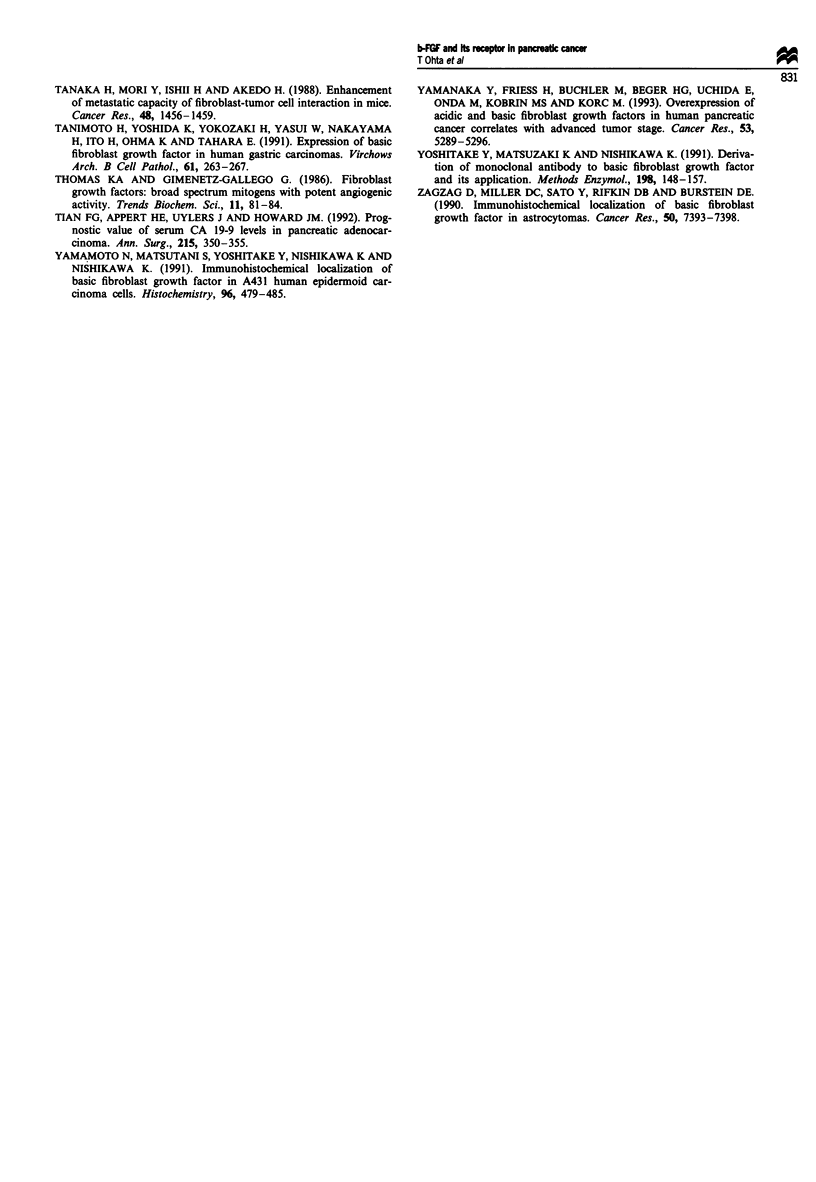


## References

[OCR_00688] Alanen K. A., Joensuu H., Klemi P. J., Nevalainen T. J. (1990). Clinical significance of nuclear DNA content in pancreatic carcinoma.. J Pathol.

[OCR_00693] Ariyama J., Suyama M., Ogawa K., Ikari T., Nagaiwa J., Fujii D., Tsuchida A. (1990). The detection and prognosis of small pancreatic carcinoma.. Int J Pancreatol.

[OCR_00701] Auron P. E., Webb A. C., Rosenwasser L. J., Mucci S. F., Rich A., Wolff S. M., Dinarello C. A. (1984). Nucleotide sequence of human monocyte interleukin 1 precursor cDNA.. Proc Natl Acad Sci U S A.

[OCR_00706] Beauchamp R. D., Lyons R. M., Yang E. Y., Coffey R. J., Moses H. L. (1990). Expression of and response to growth regulatory peptides by two human pancreatic carcinoma cell lines.. Pancreas.

[OCR_00711] Becker D., Meier C. B., Herlyn M. (1989). Proliferation of human malignant melanomas is inhibited by antisense oligodeoxynucleotides targeted against basic fibroblast growth factor.. EMBO J.

[OCR_00715] Coucke P., De Leval L., Leyh P., Bonjean K., Siwek B., Noel A., De Pauw-Gillet M. C., Paulus J. M., Bassleer R., Foidart J. M. (1992). Influence of laminin or fibroblasts upon colony formation in the mouse by B16 melanoma cell spheroids: a morphometric analysis.. In Vivo.

[OCR_00722] Eguchi J., Nomata K., Kanda S., Igawa T., Taide M., Koga S., Matsuya F., Kanetake H., Saito Y. (1992). Gene expression and immunohistochemical localization of basic fibroblast growth factor in renal cell carcinoma.. Biochem Biophys Res Commun.

[OCR_00732] Ensoli B., Nakamura S., Salahuddin S. Z., Biberfeld P., Larsson L., Beaver B., Wong-Staal F., Gallo R. C. (1989). AIDS-Kaposi's sarcoma-derived cells express cytokines with autocrine and paracrine growth effects.. Science.

[OCR_00736] Folkman J., Klagsbrun M. (1987). Angiogenic factors.. Science.

[OCR_00738] Friesel R., Burgess W. H., Mehlman T., Maciag T. (1986). The characterization of the receptor for endothelial cell growth factor by covalent ligand attachment.. J Biol Chem.

[OCR_00751] Gospodarowicz D., Neufeld G., Schweigerer L. (1987). Fibroblast growth factor: structural and biological properties.. J Cell Physiol Suppl.

[OCR_00746] Gärtner M. F., Wilson E. L., Dowdle E. B. (1992). Fibroblast-dependent tumorigenicity of melanoma xenografts in athymic mice.. Int J Cancer.

[OCR_00754] Hughes S. E., Hall P. A. (1993). Immunolocalization of fibroblast growth factor receptor 1 and its ligands in human tissues.. Lab Invest.

[OCR_00764] Kayahara M., Nagakawa T., Ueno K., Ohta T., Takeda T., Miyazaki I. (1993). An evaluation of radical resection for pancreatic cancer based on the mode of recurrence as determined by autopsy and diagnostic imaging.. Cancer.

[OCR_00775] Klagsbrun M., Baird A. (1991). A dual receptor system is required for basic fibroblast growth factor activity.. Cell.

[OCR_00782] Kobrin M. S., Yamanaka Y., Friess H., Lopez M. E., Korc M. (1993). Aberrant expression of type I fibroblast growth factor receptor in human pancreatic adenocarcinomas.. Cancer Res.

[OCR_00800] Li V. W., Folkerth R. D., Watanabe H., Yu C., Rupnick M., Barnes P., Scott R. M., Black P. M., Sallan S. E., Folkman J. (1994). Microvessel count and cerebrospinal fluid basic fibroblast growth factor in children with brain tumours.. Lancet.

[OCR_00805] March C. J., Mosley B., Larsen A., Cerretti D. P., Braedt G., Price V., Gillis S., Henney C. S., Kronheim S. R., Grabstein K. (1985). Cloning, sequence and expression of two distinct human interleukin-1 complementary DNAs.. Nature.

[OCR_00812] Mason I. J. (1994). The ins and outs of fibroblast growth factors.. Cell.

[OCR_00814] Matsuzaki K., Yoshitake Y., Matuo Y., Sasaki H., Nishikawa K. (1989). Monoclonal antibodies against heparin-binding growth factor II/basic fibroblast growth factor that block its biological activity: invalidity of the antibodies for tumor angiogenesis.. Proc Natl Acad Sci U S A.

[OCR_00823] Miyamoto M., Naruo K., Seko C., Matsumoto S., Kondo T., Kurokawa T. (1993). Molecular cloning of a novel cytokine cDNA encoding the ninth member of the fibroblast growth factor family, which has a unique secretion property.. Mol Cell Biol.

[OCR_00830] Morohoshi T., Kanda M., Asanuma K., Klöppel G. (1989). Intraductal papillary neoplasms of the pancreas. A clinicopathologic study of six patients.. Cancer.

[OCR_00833] Motojima K., Tsunoda T., Kanematsu T., Nagata Y., Urano T., Shiku H. (1991). Distinguishing pancreatic carcinoma from other periampullary carcinomas by analysis of mutations in the Kirsten-ras oncogene.. Ann Surg.

[OCR_00839] Nakamori S., Ishikawa O., Ohigashi H., Imaoka S., Sasaki Y., Kameyama M., Kabuto T., Furukawa H., Iwanakga T., Kimura N. (1993). Clinicopathological features and prognostic significance of nucleoside diphosphate kinase/nm23 gene product in human pancreatic exocrine neoplasms.. Int J Pancreatol.

[OCR_00850] Nakanishi Y., Kihara K., Mizuno K., Masamune Y., Yoshitake Y., Nishikawa K. (1992). Direct effect of basic fibroblast growth factor on gene transcription in a cell-free system.. Proc Natl Acad Sci U S A.

[OCR_00853] Ohta T., Nagakawa T., Ueno K., Kayahara M., Mori K., Kobayashi H., Takeda T., Miyazaki I. (1993). The mode of lymphatic and local spread of pancreatic carcinomas less than 4.0 cm in size.. Int Surg.

[OCR_00859] Olwin B. B., Hauschka S. D. (1989). Cell type and tissue distribution of the fibroblast growth factor receptor.. J Cell Biochem.

[OCR_00866] Ristow H. J., Messmer T. O. (1988). Basic fibroblast growth factor and insulin-like growth factor I are strong mitogens for cultured mouse keratinocytes.. J Cell Physiol.

[OCR_00871] Rizzino A., Ruff E., Rizzino H. (1986). Induction and modulation of anchorage-independent growth by platelet-derived growth factor, fibroblast growth factor, and transforming growth factor-beta.. Cancer Res.

[OCR_00877] Satake K., Chung Y. S., Umeyama K., Takeuchi T., Kim Y. S. (1991). The possibility of diagnosing small pancreatic cancer (less than 4.0 cm) by measuring various serum tumor markers. A retrospective study.. Cancer.

[OCR_00884] Schweigerer L., Neufeld G., Friedman J., Abraham J. A., Fiddes J. C., Gospodarowicz D. (1987). Capillary endothelial cells express basic fibroblast growth factor, a mitogen that promotes their own growth.. Nature.

[OCR_00890] Schweigerer L., Neufeld G., Mergia A., Abraham J. A., Fiddes J. C., Gospodarowicz D. (1987). Basic fibroblast growth factor in human rhabdomyosarcoma cells: implications for the proliferation and neovascularization of myoblast-derived tumors.. Proc Natl Acad Sci U S A.

[OCR_00897] Takahashi J. A., Mori H., Fukumoto M., Igarashi K., Jaye M., Oda Y., Kikuchi H., Hatanaka M. (1990). Gene expression of fibroblast growth factors in human gliomas and meningiomas: demonstration of cellular source of basic fibroblast growth factor mRNA and peptide in tumor tissues.. Proc Natl Acad Sci U S A.

[OCR_00904] Tanaka A., Miyamoto K., Minamino N., Takeda M., Sato B., Matsuo H., Matsumoto K. (1992). Cloning and characterization of an androgen-induced growth factor essential for the androgen-dependent growth of mouse mammary carcinoma cells.. Proc Natl Acad Sci U S A.

[OCR_00915] Tanaka H., Mori Y., Ishii H., Akedo H. (1988). Enhancement of metastatic capacity of fibroblast-tumor cell interaction in mice.. Cancer Res.

[OCR_00920] Tanimoto H., Yoshida K., Yokozaki H., Yasui W., Nakayama H., Ito H., Ohama K., Tahara E. (1991). Expression of basic fibroblast growth factor in human gastric carcinomas.. Virchows Arch B Cell Pathol Incl Mol Pathol.

[OCR_00933] Tian F., Appert H. E., Myles J., Howard J. M. (1992). Prognostic value of serum CA 19-9 levels in pancreatic adenocarcinoma.. Ann Surg.

[OCR_00938] Yamamoto N., Matsutani S., Yoshitake Y., Nishikawa K. (1991). Immunohistochemical localization of basic fibroblast growth factor in A431 human epidermoid carcinoma cells.. Histochemistry.

[OCR_00944] Yamanaka Y., Friess H., Buchler M., Beger H. G., Uchida E., Onda M., Kobrin M. S., Korc M. (1993). Overexpression of acidic and basic fibroblast growth factors in human pancreatic cancer correlates with advanced tumor stage.. Cancer Res.

[OCR_00949] Yoshitake Y., Matsuzaki K., Nishikawa K. (1991). Derivation of monoclonal antibody to basic fibroblast growth factor and its application.. Methods Enzymol.

[OCR_00954] Zagzag D., Miller D. C., Sato Y., Rifkin D. B., Burstein D. E. (1990). Immunohistochemical localization of basic fibroblast growth factor in astrocytomas.. Cancer Res.

